# The association between urinary genistein levels and mortality among adults in the United States

**DOI:** 10.1371/journal.pone.0211368

**Published:** 2019-01-25

**Authors:** Carolyn Marcelo, Melissa Warwick, Catherine Marcelo, Rehan Qayyum

**Affiliations:** Division of Hospital Medicine, Department of Internal Medicine, Virginia Commonwealth University School of Medicine, Richmond, Virginia, United States of America; Weill Cornell Medical College Qatar, QATAR

## Abstract

**Background:**

Current research on the relationship between phytoestrogens and mortality has been inconclusive. We explored the relationship between genistein, a phytoestrogen, and mortality in a large cohort representative of the United States population.

**Methods:**

Data were analyzed from the National Health and Nutrition Examination Survey (NHANES) from 1999–2010. Normalized urinary genistein (nUG) was analyzed as a log-transformed continuous variable and in quartiles. Mortality data were obtained from the National Death Index and matched to the NHANES participants. Survival analyses were conducted using the Kaplan-Meier analysis. Cox proportional hazard models were constructed for all-cause and cause-specific mortality without and with adjustment for potential confounding variables.

**Results:**

Of 11,497 participants, 944 died during the 64,443 person-years follow-up. The all-cause mortality rate was significantly lower in the lowest quartile compared to the highest quartile (incidence rate ratio = 2.14, 95%CI = 1.76 to 2.60). Compared to the lowest quartile, the highest quartile had significantly higher adjusted all-cause (HR = 1.57, 95%CI = 1.23 to 2.00), cardiovascular (HR = 1.67, 95%CI = 1.04 to 2.68), and other-cause (HR = 1.85, 95%CI = 1.33 to 2.57) mortality.

**Conclusion:**

We found that high urinary genistein levels were associated with increased risk of all-cause, cardiovascular, and other-cause mortality. This is contrary to popular opinion on the health benefits of genistein and needs further research.

## Introduction

Phytoestrogens are plant-based chemical compounds with structural similarities to female sex hormones such as 17-β-estradiol and diethylstilbestrol [1]. The wide range of naturally-occurring phytoestrogens can be sub-classified into several groups: isoflavones, prenylflavonoids, coumestans, and lignans. The most common phytoestrogens are isoflavones found in a variety of fruits and vegetables, particularly soy products, legumes, and flax [2]. One of the major isoflavones that is commonly consumed by humans is genistein, which is typically found in soy products [1].

Several studies have suggested that phytoestrogens are associated with a lower risk of all-cause mortality [3,4], cardiovascular disease mortality [3], and cancer mortality [5–8]. Other studies have found no benefit of phytoestrogen intake on all-cause, cardiovascular, or cancer mortality [9–14]. Interestingly, some studies have found an increased mortality risk with phytoestrogen intake [15,16]. The conflicting results from these studies may, at least partly, be due to participant self-reports of dietary phytoestrogen intake—a measure associated with significant error in the measurement of actual phytoestrogen intake [17].

Urinary excretion of phytoestrogen is an objective measure which is independent of recall bias associated with self-reports of dietary intake. Several studies have found that urine isoflavone concentrations were significantly correlated to dietary intake [18–20]. Given the conflicting results of prior studies, we decided to explore the relationship between genistein, a phytoestrogen, and all-cause, cardiovascular, cancer, and other-cause mortality in a large cohort representative of the United States population using urinary genistein levels as a marker of dietary intake.

## Methods

We used the continuous National Health and Nutrition Examination Survey (NHANES), an ongoing cross-sectional, complex, multistage, stratified, clustered sampling design survey representative of the civilian, noninstitutionalized population of the United States. NHANES was approved by the Centers for Disease Control and Prevention’s Institutional Review Board, and all participants provided written informed consent. Detailed descriptions of survey methods and procedures are available on the NHANES website [21]. Participants from six survey cycles (1999–2010) who were older than 20 years were included in the study. Mortality data were obtained from the National Death Index (NDI), which is a centralized database of death records. The National Center for Health Statistics linked surveys to death records from the NDI by matching identifying information such as Social Security number. We categorized cause of deaths into all-cause deaths, cardiovascular deaths, deaths due to cancer, and deaths due to other causes (other-cause mortality).

### Study variables

Race, sex, and age were self-reported, and all ages greater than 85 years were recorded as 85. Young was defined as age 45 years or younger, middle-age was defined as ages younger than 65 years and older than 45 years, and elderly was defined as age 65 years and older. Subjects were categorized as active smokers, ever-smokers who had smoked at least 100 cigarettes in their lifetime, or nonsmokers who had never smoked. The body mass index (BMI) was calculated by dividing the weight in kilograms by the height in meters squared, with height and weight obtained by trained body measurement health technicians. Hypertension was defined as carrying a diagnosis of hypertension, use of antihypertensive medications, or having an average of three blood pressure measurements greater than 140/90. Blood pressure was measured by certified blood pressure examiners who averaged three to four manual blood pressure measurements. Diabetes was categorized as participants who had ever been diagnosed with diabetes, had a hemoglobin A1C greater than 7.0%, or were on medication for diabetes. Hemoglobin A1C was measured on the fully automated glycohemoglobin analyzer. C-reactive protein (CRP) was measured from serum samples by latex-enhanced nephelometry. Cholesterol was measured in serum via the Roche Modular P chemistry analyzer (Roche Diagnostics, Indianapolis, Indiana, USA) using an enzymatic process. Serum creatinine was measured using the Jaffe rate method (kinetic alkaline picrate). Glomerular filtration rate (GFR) was estimated using the Chronic Kidney Disease Epidemiology Collaboration (CKD-EPI) equation [22]. Urinary creatinine was measured using the Roche/Hitachi Modular P Chemistry Analyzer (Roche Diagnostics, Indianapolis, Indiana, USA) at the University of Minnesota. Glucuronidated genistein in human urine samples was deconjugated enzymatically, separated from other urine components by reversed phase high performance liquid chromatography, detected by atmospheric pressure photoionization-tandem mass spectrometry, and quantified by isotope dilution. Urinary genistein concentrations were normalized for urine dilution or concentration using urinary creatinine (urinary genistein concentration in ng/mL divided by urinary creatinine concentration in mg/dL).

### Statistical methods

Mean (SD), median (interquartile range), or frequencies were used as descriptive statistics as appropriate. We conducted all analyses while taking into account the complex survey sampling design of the NHANES. Survey weights were generated from the six 2-year cycles for the complete dataset so that the results are representative of the United States population. Survival estimates were obtained using Kaplan-Meier method. Cox proportional hazard models were constructed with and without adjustment for sex, race, hypertension, diabetes mellitus, and smoking (categorical variables); and age, BMI, serum CRP, cholesterol, and estimated GFR (continuous variables). We conducted all-cause mortality and cause-specific mortality analyses as outcomes. We log-transformed normalized urinary genistein levels due to distribution skewness and analyzed it as a continuous variable. To confirm that the observed relationship is independent of the statistical assumption about the distribution of normalized urinary genistein (nUG), we also examined adjusted and unadjusted models with quartiles of nUG. Test for trend was performed by entering the categorical variable of quartiles as continuous variable in the model. For sensitivity analysis, we used competing-risk models to confirm that the relationship we found was independent of the assumptions of the hazard distributions. All analyses were conducted using Stata 14.0 (College Station, Texas, USA).

## Results

### Descriptive results

Of the 11,497 participants in the study, 52% were female, 23% were elderly, 49% were white, 36% had hypertension, 12% had diabetes, and 21% were current smokers (Table 1). The mean (SD) age was 49.2 (18.4) years, BMI was 28.8 (6.6) kg/m^2^, and GFR was 93.7 (24.2) ml/min. The median (IQR) serum CRP was 0.21 (0.42) mg/dL and nUG was 0.24 (0.73) ng/mL/mg creatinine. During the mean follow-up of 5.6 (3.6) years and total follow-up of 64,443 person-years, there were 944 deaths—231 deaths due to cardiovascular disease, 239 deaths due to malignancy, and 474 deaths due to other causes.

**Table 1:** Population variables by mortality status.

Variable	Mortality Status			P-value
	Deceased (N = 944)	Alive (N = 10,553)	Total (N = 11,497)	
Normalized urinary genistein (ng/mL/mg creatinine)*	0.33 (1.13)	0.22 (0.71)	0.23 (0.73)	<0.001
Age (years)	70.9 (13.79)	47.2 (17.57)	49.2 (18.48)	<0.001
Young	63 (6.67)	5295 (50.18)	5358	<0.001
Middle Age	195 (20.66)	3263 (30.92)	3458	<0.001
Elderly	686 (72.67)	1995 (18.90)	2681	<0.001
BMI (kg/m^2^)	27.7 (5.9)	28.9 (6.6)	28.8	<0.001
Estimated GFR (mL/min/1.73 m2)	69.2 (23.5)	95.8 (23.1)	93.7 (24.2)	<0.001
CRP (mg/dL)*	0.32 (0.67)	0.20 (0.40)	0.21 (0.3)	<0.001
Cholesterol (mg/dL)	200.6 (43.2)	198.5 (42.3)	198.7 (42.4)	0.16
Creatinine (mg/dL)	1.10 (0.62)	0.87 (0.29)	0.89 (0.34)	<0.001
Female	418 (44.28)	5608 (53.14)	6026	<0.001
Hypertension	614 (65.04)	3518 (33.34)	4132	<0.001
Diabetes	220 (23.31)	1170 (11.09)	1390	<0.001
**Race**				
Black	173 (18.33)	2021 (19.15)	2194	<0.001
Hispanic	190 (20.13)	3003 (28.46)	3193	<0.001
White	561 (59.43)	5055 (47.90)	5616	<0.001
Other race	20 (2.12)	474 (4.49)	494	<0.001
**Tobacco Use**				
Current smoker	207 (21.93)	2223 (21.08)	2430	<0.001
Ever smoker	368 (38.98)	2544 (24.13)	2912	<0.001
Nonsmoker	369 (39.09)	5777 (54.79)	6146	<0.001

Data are presented as mean (SD) for continuous variables and N (%) for categorical variables. P-values were generated by X^2^ test or two-sample t test as indicated.

*Variable presented as median (interquartile range). P-values were generated by the Kruskal-Wallis test.

### All-cause mortality

**Fig 1:**
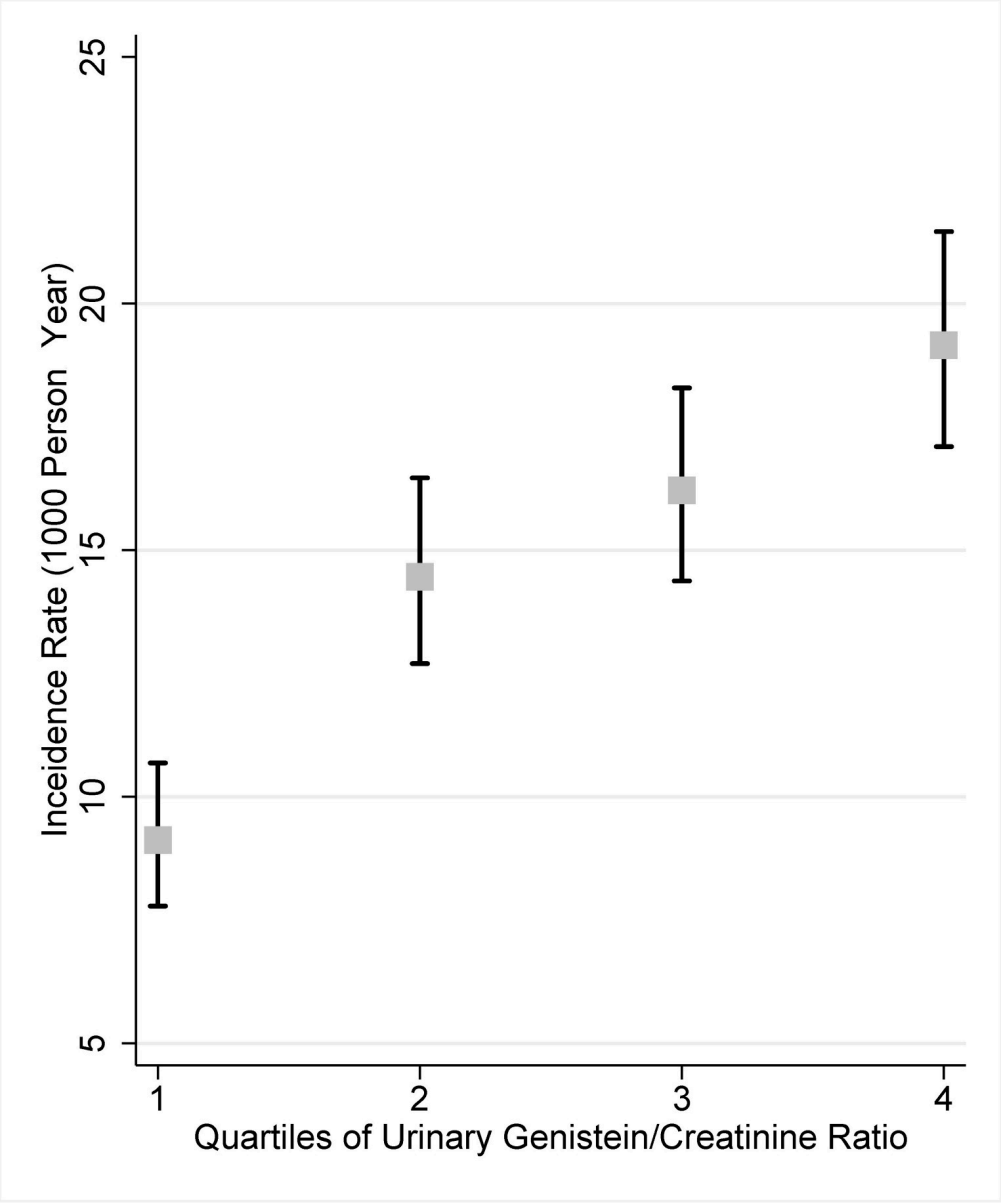
Mortality incidence rates (per 1000 person-years) for each quartile of normalized urinary genistein levels. The incidence rate of death was significantly lower (p < 0.001) in the first quartile of normalized urinary genistein (nUG) compared to the fourth quartile of nUG. The mortality incidence rate was 9.1 deaths per 1000 person-years (CI 7.8 to 10.7) in the first quartile versus 19.2 deaths per 1000 person years (CI 17.1 to 21.5) in the fourth quartile.

**Fig 2:**
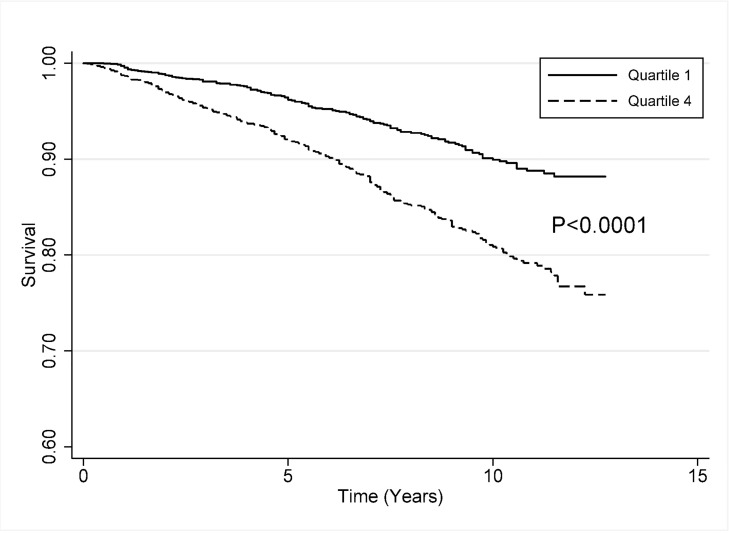
Kaplan-Meier curve comparing survival rates of the lowest and highest quartiles of normalized urinary genistein. There was a statistically significant higher survival rate among individuals in the first quartile of normalized urinary genistein (nUG) compared to those in the fourth quartile of nUG (p < 00001).

The mortality rate was 9.1 (95%CI = 7.8 to 10.7) deaths per 1000 person-years in the lowest quartile compared to 19.2 (95%CI = 17.1 to 21.5) deaths per 1000 person-years in the highest quartile (incidence rate ratio = 2.14, 95%CI = 1.76 to 2.60, p < 0.001) (Fig 1). Using the Kaplan-Meier analysis, survival was statistically significantly higher (p< 0.0001) in the lowest quartile of nUG than in the highest quartile–there were 153 deaths in the lowest quartile and 298 deaths in the highest quartile during the study time span (Fig 2).

**Fig 3:**
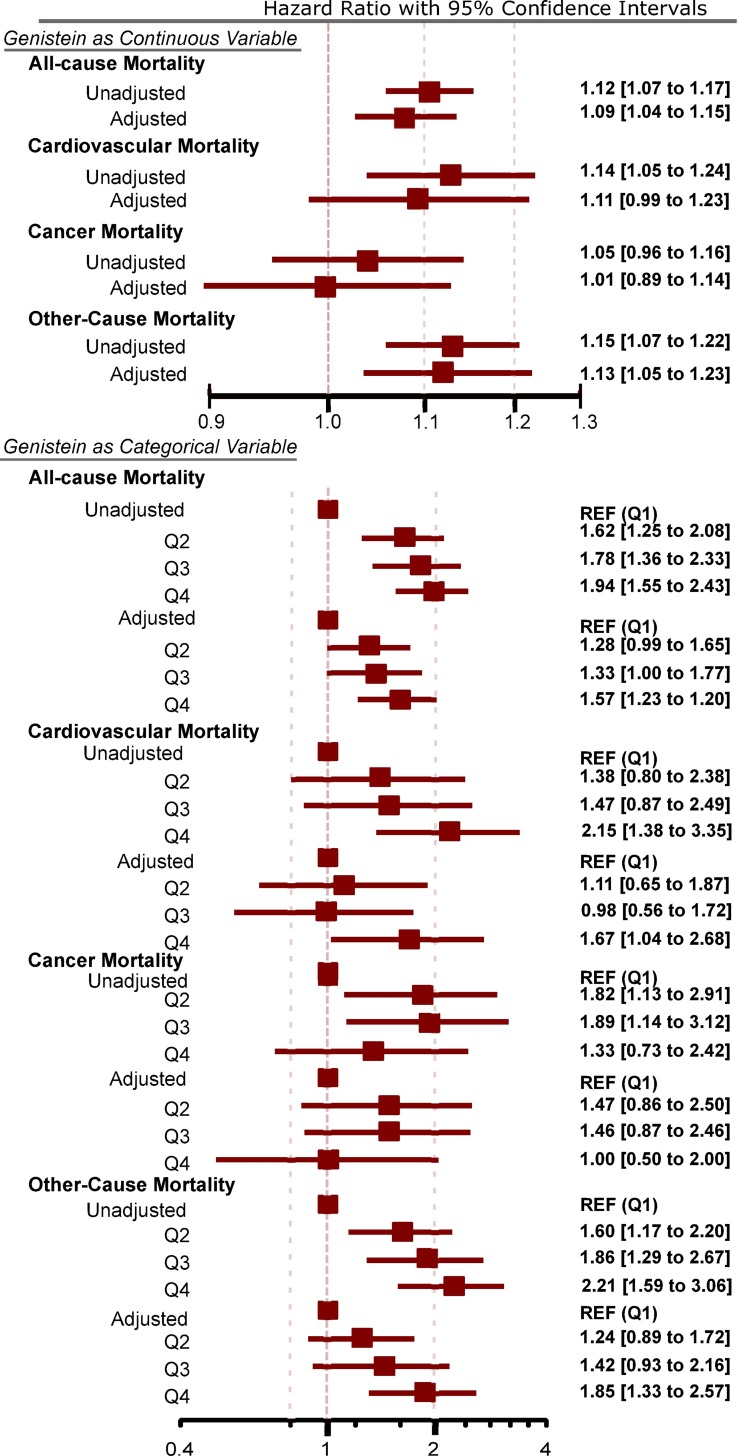
Adjusted and unadjusted all-cause and cause-specific mortality. Using Cox proportional hazard models, there was a statistically significant increase in all-cause mortality with higher levels of nUG in both adjusted and unadjusted data. There was a statistically significant increase in cardiovascular mortality when examining quartiles of nUG (both adjusted and unadjusted models) but not when nUG was examined as a continuous variable. There was no statistically significant relationship between nUG and cancer mortality in either adjusted or unadjusted data. Finally, there was a statistically significant increase in other-cause mortality with higher levels of nUG in both adjusted and unadjusted data.

Using the Cox proportional hazards model, we found a statistically significant direct relationship between nUG levels and risk of mortality that was robust when adjusting for confounders (Fig 3). In adjusted models, for every log increase in nUG, there was a 9% increase in risk of mortality (HR = 1.09, 95%CI = 1.04 to 1.15, p = 0.001), and individuals in the highest quartile of nUG had a 57% higher risk of mortality compared to individuals in the lowest quartile (HR = 1.57, 95%CI = 1.23 to 2.00, p < 0.0001). Furthermore, the trend for increasing mortality was significant without (HR = 1.21, 95%CI = 1.13 to 1.30, p < 0.001) and with adjustment (HR = 1.14, 95%CI = 1.0 to 1.24, p = 0.001) for potential confounders.

### Cause-specific mortality

Using the Cox proportional hazards model, for every log increase of nUG, there was a 14% increase in risk of cardiovascular disease mortality (HR = 1.14, 95%CI = 1.05 to 1.24, p = 0.002) (Fig 3). However, after adjusting for potential confounders, the relationship between nUG and the risk for cardiovascular disease mortality did not reach statistical significance (HR = 1.11, 95%CI = 0.99 to 1.24, p = 0.06). When analyzing data using quartiles of nUG, however, the relationship was statistically significant in unadjusted and adjusted analyses. In adjusted models, individuals in the highest quartile of nUG had a 67% higher risk of cardiovascular disease mortality compared to individuals in the lowest quartile (HR = 1.67, 95%CI = 1.04 to 2.68, p = 0.03).

For cancer mortality (Fig 3), there was no statistically significant relationship with nUG without adjustment (HR = 1.05, 95%CI = 0.96 to 1.16, p = 0.28) and with adjustment (HR = 1.01, 95%CI = 0.89 to 1.14, p = 0.87) for potential confounders. Similarly, comparing the lowest to the highest quartile of nUG, there was no statistically significant relationship in unadjusted (HR = 1.33, 95%CI = 0.73 to 2.42, p = 0.35) or adjusted models (HR = 1.01, 95%CI = 0.51 to 2.00, p = 0.99).

Finally, there was a statistically significant direct relationship between nUG and other-cause mortality in unadjusted and adjusted analyses (Fig 3). Using adjusted data, every log increase in nUG was associated with a 13% increase in risk of other-cause mortality (HR = 1.13, 95%CI = 1.05 to 1.23, p = 0.002). Similarly, we found a significantly higher risk of other-cause mortality in participants in the highest than in the lowest quartile in unadjusted (HR = 2.21, 95%CI = 1.59 to 3.06, p < 0.001) and adjusted models (HR = 1.85, 95%CI = 1.33 to 2.57, p < 0.0001).

### Sensitivity analysis

Competing risk models for cardiovascular, cancer, and other-cause mortality found results similar to the Cox proportional hazards model. For cardiovascular mortality, the association seen in unadjusted analysis (HR = 1.15, 95%CI = 1.08 to 1.24, p < 0.0001) disappeared after adjusting for potential confounders (HR = 1.08, 95%CI = 1.00 to 1.18, p = 0.06). As compared to the lowest quartile, participants in the highest quartile had higher cardiovascular mortality risk in unadjusted (HR = 2.4, 95%CI = 1.60 to 3.60, p < 0.001) and adjusted analyses (HR = 1.79, 95%CI = 1.15 to 2.79, p = 0.01). For cancer mortality, unadjusted and adjusted results remained non-significant with nUG as continuous variable or in quartiles. Similar to the Cox proportional hazards models, other-cause mortality was significantly associated with nUG. In adjusted models, every log increase in nUG was associated with 13% higher mortality risk (HR = 1.13, 95%CI = 1.06 to 1.20, p < 0.0001), and individuals in the highest quartile had a 70% higher risk of other-cause mortality than those in the lowest quartile (HR = 1.70, 95%CI = 1.27 to 2.27, p < 0.0001).

## Discussion

In this study, we report an association between higher urinary genistein levels and increased risk of all-cause and other-cause mortality that was independent of potential confounding variables and robust to distributional assumptions of the nUG. We further found that nUG has no association with cancer mortality. The association between nUG and cardiovascular mortality was complex; the association was independent of potential confounding variables when nUG was examined in quartiles but not when examined as a log-transformed continuous variable. Our results were robust to statistical modeling assumptions and remained unchanged in sensitivity analyses using competing-risk models.

Genistein is quickly absorbed after oral intake due to its small molecular weight and lipophilic properties. It has a low oral bioavailability due to its high rate of metabolism in the intestine and liver. Remaining genistein metabolites move to the liver via the portal vein and undergoes further metabolism. Genistein is mostly distributed in the gastrointestinal tract and liver due to its hydrophilic nature preventing its diffusion and accumulation in other tissues. Additionally, genistein undergoes enterohepatic recycling via highly active efflux transporters in the intestine which excrete genistein back into the lumen of the gastrointestinal tract where it is metabolized by gut flora and reabsorbed by the enterocytes. However, genistein metabolites can also be found in the urine in moderate amounts which suggests that urinary excretion is one of the major elimination pathways of genistein [23]. Individual differences in enzymatic activity within the intestine and liver as well as gut microbiome may result in differences in how genistein is metabolized which may in turn affect the amount of genistein excreted in the urine.

Several molecular pathways may be responsible for the observed findings between high urinary genistein levels and increased mortality. Genistein may have a potentially deleterious effect on the immune system as it decreases thymic size in ovariectomized female and male mice in a dose-dependent manner. The effect on immune system may be independent of its interaction with estrogen receptor as an estrogen receptor blocker did not completely block genistein’s effect on thymic size [24]. Genistein’s effect on the thymus may lead to splenic and total body lymphocytopenia through inducing apoptosis of CD4+/CD8+ and CD4+/CD8- thymocytes [24]. Furthermore, genistein has also been shown to decrease delayed hypersensitivity responses [25]. Thus, genistein may have significant implications for humoral and cellular immunity. Genistein may also cause genotoxicity by increasing DNA breakage through its effect on the topoisomerase II enzyme [26,27]. In fact, genistein genotoxicity has been observed in a wide variety of cell lines including Chinese hamster lung fibroblasts, L5178Y mouse lymphoma cells, and human colon and lymphocyte cell lines [28–32].

Few epidemiological studies have examined the relationship between all-cause or cause-specific mortality and phytoestrogens in general, or genistein specifically, with conflicting results. A systematic review of phytoestrogens’ association with all-cause mortality included eight studies that collectively enrolled 97,118 individuals and identified 12,228 deaths during a median follow-up of 12.8 years [33]. The systematic review found a pooled relative risk of 0.86 (95%CI = 0.73 to 1.00) although there was significant heterogeneity between the studies (I^2^ = 68%; P = 0.003). For the association of phytoestrogen intake and cardiovascular mortality, this systematic review found 13 studies including 338,541 participants and 7774 deaths and reported a pooled relative risk of 0.86 (95%CI = 0.75 to 0.98) [33]. Of note, studies included in this review measured phytoestrogen intake using self-report through various methods [33]. Another systematic review identified two studies that measured phytoestrogen intake using more objective measures such as spot urine or serum levels and examined the association between phytoestrogen intake and all-cause mortality. These studies had conflicting results for phytoestrogens as a group but found no association of genistein with all-cause mortality; results from these two studies were not pooled [34].

Reger, et al performed a similar study in 2014 reviewing NHANES data from 1999–2004. They found that the median urinary concentrations of total isoflavones were higher in study participants who died from cardiovascular disease suggesting an increased risk of cardiovascular mortality is associated with higher concentration of urinary isoflavones [16]. Their study examined multiple isoflavones as a group, including genistein, daidzein, equol, and O-desmethylangolensin, and although their findings were significant in isoflavones as a group and in daidzein individually, they did not report significant findings in urinary genistein levels. This subtle difference in their findings from our study may be attributed to a lower sample size. While this study also examined NHANES data, they used data only from 1999 through 2004 while our study examined NHANES data from 1999 through 2010. We therefore had a much larger study population (11,753 in our study, compared to 5,179 in their study) and longer follow-up time to allow more time for change. Additionally, we adjusted for more known risk factors associated with cardiovascular disease, including diabetes mellitus and hypertension, which was not included in Reger, et al’s analysis.

Most studies examining the relationship between phytoestrogens and mortality were conducted in breast cancer patients. Several of these studies showed an inverse relationship between phytoestrogens and mortality from breast cancer [5–7]. In addition, all-cause mortality was found to be lower in women with ER-negative/PR-negative breast cancer and high intake of isoflavones in one study enrolling a diverse cohort [8]. In contrast, several other studies found no association between phytoestrogen intake and mortality [6,15,35]. When phytoestrogen intake was further categorized by the group of phytoestrogens in one study, only high lignan intake was associated with lower risk of all-cause mortality in post-menopausal breast cancer patients [15]. Consistent with most other studies noted above, these studies relied on self-report by patients to quantify dietary phytoestrogens intake. Three studies that examined serum levels of a phytoestrogen, enterolactone, found that high serum enterolactone levels were associated with significant reduction in all-cause mortality and breast cancer-specific mortality in breast cancer patients [36–38].

The conflicting results above may arise from the recall bias and measurement error that is inherent in self-report methods of determining dietary intake. In addition, it is possible that phytoestrogens may not have a specific group-effect on all-cause or cause-specific mortality and each member of the phytoestrogen group needs to be examined separately. To this end, we examined a more objective method of measurement thus eliminating recall bias and possibly reducing measurement error. Furthermore, we focused on an individual member of the phytoestrogen group and determined the effect of genistein.

Our study has important clinical, public health, and research implications. Findings from this study are contrary to the perceived benefits of phytoestrogens and soy-rich diets. Given the observed association of an increase in mortality with increase in urinary genistein levels, the safety of genistein needs to be examined before any clinical recommendation on its use in practice. Soy-products are rich in genistein and are commonly used in various dietary supplements, suggesting a public health urgency for a better understanding. As the public continues to focus on potential benefits of genistein and other phytoestrogens, the use of these substances in health supplements may continue to increase. Thus, there is a growing need for more research examining the long-term effects and safety of genistein. Further research is needed to identify which, if any, of the phytoestrogens promote or protect health. In addition, molecular mechanisms through which genistein acts need to be explored further.

This study had several strengths and limitations. The main strengths of this study are the use of a diverse large cohort representative of the United States population and the use of hard endpoints (mortality). Additionally, in our analyses we accounted for competing events, i.e. cause of mortality, that was not accounted for in the Kaplan-Meier and Cox proportional hazard models by performing a competing risk analysis, which confirmed our findings in the other models. Urinary genistein has been positively correlated to dietary intake in previous studies [18–20] and therefore could be considered an objective measure of dietary intake, but individual differences in metabolism could potentially affect urinary genistein levels and therefore affect this correlation with dietary intake. Additionally, since this is an observational study, we cannot determine if there is a causal relationship between genistein levels and mortality. Further, urinary genistein levels were measured at one time point only limiting our ability to assess changes in genistein intake.

In conclusion, we found an increased risk of all-cause mortality with higher levels of urinary genistein. The mechanisms by which genistein may increase mortality is uncertain and warrants further study. Given the findings of this study, the long-term effects and safety of genistein will need to be further investigated.
